# High Hydrovoltaic Power Density Achieved by Universal Evaporating Potential Devices

**DOI:** 10.1002/advs.202302941

**Published:** 2023-09-15

**Authors:** Fei Yu, Jialun Li, Yi Jiang, Liying Wang, Xijia Yang, Yue Yang, Xuesong Li, Ke Jiang, Wei Lü, Xiaojuan Sun

**Affiliations:** ^1^ Key Laboratory of Advanced Structural Materials, Ministry of Education & Advanced Institute of Materials Science Changchun University of Technology Changchun 130012 P.R. China; ^2^ School of Science Changchun Institute of Technology Changchun 130012 P. R. China; ^3^ State Key Laboratory of Luminescence and Applications, Changchun Institute of Optics, Fine Mechanics and Physics Chinese Academy of Sciences Changchun 130033 P. R. China

**Keywords:** energy storage, evaporating potential, hydrovoltaic effect, power density

## Abstract

While hydrovoltaic electrical energy generation developments in very recent years have provided an alternative strategy to generate electricity from the direct interaction of materials with water, the two main issues still need to be addressed: achieving satisfactory output power density and understanding the reliable mechanism. In the present work, the integration of capacitors and water evaporation devices is proposed to provide a stable power supply. The feasible device structure consuming only water and air is green and environmentally sustainable, achieving a recorded power density of 142.72 µW cm^−2^. The output power of the series of devices is sufficient to drive portable electronic products with different voltage and current requirements, enabling self‐driving systems for portable appliances. It has been shown that the working behavior originates from evaporating potential other than streaming potential. The present work provides both theoretical support and an experimental design for realizing practical application of hydrovoltaic electrical energy generation devices.

## Graphical abstract

The electrons on the carbon cloth (CC) surface are transferred to the H_2_O molecule, and when the H_2_O molecule evaporates, the electrons instantaneously return to the CC surface to be absorb by the H_2_O and O_2_, generating OH^‐^, which forms an electric current under the action of the electric field forces.

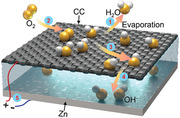

## 1 Introduction

Energy, fresh water, and food are basic necessities for mankind surviving.^[^ ^1^ ^]^ The development of IT technology and the increasing global population further pull up the consumption of energy, inducing a probable crisis for a stable supply of fresh water and food.^[^ ^2^ ^]^ While numerous clean and sustainable energies have been suggested and developed, they are currently limited by unsatisfied efficiency and high cost. A very recently suggested conception, “Hydrovoltaic effect”, has provided an alternative strategy to generate electricity from the direct interaction of materials with water.^[^ ^3^ ^]^

Quincke generated electricity from water by direct interaction between water and solid, in a process by which a flow of electrolytes under a pressure gradient through a narrow channel generates an electric voltage in the flow, which is called streaming potential, and this process was based on electrokinetic theory.^[^ ^4^ ^]^ S. Ghosh et al. studied flow‐induced voltage and current generation in carbon nanotubes, indicating that the flow‐induced asymmetry of the random fluctuations is key to the charge‐carrier drift (drag) mechanism.^[^ ^5^ ^]^ Youn et al. mentioned that in the hydrovoltic device, the evaporation‐driven ionic (or water molecular) motions could induce the charge carrier flows through Coulombic interactions at the solid/liquid interface, which is the ionovoltaic phenomena.^[^ ^6^ ^]^ Zhou et al. mentioned that carbon nanomaterials can expose their atoms on the surfaces, permitting substantial interaction with water through electronic coupling, electricity generation in carbon nanomaterials on exposure to water flows,^[^ ^7^ ^]^ waves^[^ ^8^ ^]^ and rains,^[^ ^9^, ^10^, ^11^ ^]^ with no need of a pressure gradient as is required by the streaming potential.

In 2017, Guo et al. first reported electrical energy generation by natural evaporation through porous materials.^[^ ^12^ ^]^ Since then, the phenomenon termed as the hydrovoltaic effect has been extensively investigated and demonstrated in various porous materials.^[^ ^13^, ^14^, ^15^ ^]^ While great progress has been achieved, the two main issues still need to be addressed: understanding the reliable mechanism and achieving satisfied output power density for practical application.^[^ ^16^, ^17^, ^18^, ^19^, ^20^, ^21^ ^]^

The underlying mechanism for hydrovoltaic electrical energy generation has been considered due to flowing, waving, dropping of water molecules between the interface of solid and liquid, which is thus attributed to classical streaming potential.^[^ ^22^, ^23^ ^]^ Presently, clarified definition and explanation are still deficient since the physical behavior of liquid‐solid interaction cannot be well understood based on current theories and methods.^[^ ^24^, ^25^, ^26^, ^27^, ^28^, ^29^ ^]^ A recent report by Guo et al. in 2022 has indicated that the electricity generation by ethanol evaporation is independent of streaming potential and termed as the evaporating potential.^[^ ^30^ ^]^ As for output power density, the initial values are quite small and the recorded high output power densities are instantaneous with a typical duration of only several microseconds. Although the power density has been enhanced by greater than two orders of magnitude from an initial 100 to 60000 µW m^−2^, the further improvement is essential for commercial application.

**Figure 1:**
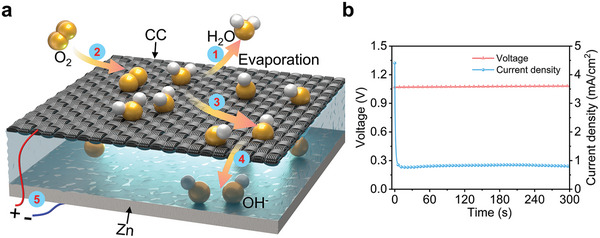
a) Schematic illustration of the device; b) the variation of voltage and current density as a function of evaporation time.

Currently, the main devices for hydrovoltaic electrical energy generation includes porous film with the bottom end inserted in bulk liquid, and the current density is limited by porous channels.^[^ ^31^ ^]^ Taking aforementioned discussion into account, we herein proposed a capacitor‐type device for hydrovoltaic electrical energy generation. The design and working mechanism is shown in **Figure** **1**. We have shown that the working behavior is consistent with evaporating potential other than streaming potential, proving that evaporating potential could also be generated in pure water system. Present work achieved followed results: i) the integration of capacitor and water evaporation device is proposed. The water evaporation process is a continuous charging process of capacitor, providing stable power supply; ii) the carriers produced by evaporation potential are efficiently utilized to produce a recorded power density of 142.72 Uw cm^−2^. As far as we know, such a simple device structure consuming only water and air is green and environmentally sustainable, and the current density and power density of the device are all recorded, enabling self‐driving systems for small appliances.

## 2 Results and Discussion

Figure 1a is schematic illustration of the device, in which Zn plate and hydrophilic carbon cloth(CC) are separated by filter paper to form a sandwiched structure. The photo of a unit device is shown in Figure S1a (Supporting Information). The mechanism can be explained as follows: In Step 1, water molecules adsorbed on the surface of the carbon material absorb environmental thermal energy and vibrate violently. Some water molecules escape from the surface of the carbon material after vibrating, realizing the process of evaporation. At the moment when water molecules escape from the carbon material, the surface charge of the carbon material is disturbed by local water molecules, which causes an increase in local electron density and excitation of surface electrons of the carbon material. The excited electrons interact with water molecules and oxygen (Step 2) on the surface of the carbon material to produce OH^−^ (Step 3), which then migrate under the help of the high electrode potential of the metal electrode and the high potential difference between the metal electrode and the carbon material (Step 4), resulting in a high current output (Step 5). The SEM images of carbon cloth are shown in Figure S1b and c (Supporting Information). The cross‐linked carbon fibers with ≈10 µm diameter enable excellent heat transfer and uniform distribution. The commercial carbon cloth is hydrophobic with 133° contact angle, which is acidified for hydrophilic treatment and the contact angle is decreased to 24^o^ as shown in Figure S2 (Supporting Information). Figure S3 (Supporting Information) is SEM images of filter paper, which is intrinsically hydrophilic with a porous structure formed by interlacing fibers. After dropping deionized water water (18.2 MΩ cm), stable voltage (1.07 V) and current density (825.54 uA cm^−2^) are detected between Zn sheet and carbon cloth as shown in Figure 1b.

**Figure 2:**
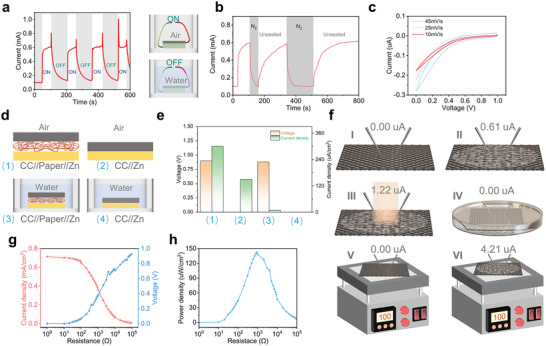
a) The j–t curve of the device with intermittent dipping in bulk water. The “on” represents exposure in air, and the “off” represents dipping in water; b) The j–t curve of the device with intermittent exposing in N_2_ and air; c) The CV curves of device; d) and e) are voltage and current density achieved with different device structure and measurement condition; f) Currents of carbon cloth measured at different condition. I: without dropping water, II: dropping water, III: dropping water and light illumination, IV: dipping in water, V: without dropping water and heated by a hot plate, VI: dropping water and heated by a hot plate; g) The variation of voltage and current density with the increasing load; h) the variation of power density with the increasing load.

**Figure** **2**a shows the j–t curve of the device with intermittent dipping in bulk water. Upon immersing the device in water, the output current of the device decreases rapidly, which is recovered upon exposing in air, showing a periodic change in current. This indicates that the output current of the device is related to water evaporation.^[^ ^32^, ^33^ ^]^ The reason for the current spikes in Figure 2a is due to external pressure, as verified in Figure S4a–d (Supporting Information). Figure 2b is the j–t curve of device with intermittent exposing in N_2_ and air. During the beginning 0–28 s, the device is covered by sealing film and the current is weak. During 28–105 s, the current recovers after removing the sealing film. After that, the device is exposed to N_2_ and air, respectively. It is obvious that the current increases in air and decreases in N_2_, indicating that oxygen is necessary for achieving current output. Figure 2c is cyclic voltammetry curves of the device measured at different scan rates. There is no redox related peak found, excluding the possibility of forming Zn‐air battery. ^[^ ^34^ ^]^

Figure 2e shows the voltage and current density achieved with different device structures as shown in Figure 2d. With the sandwiched structure and exposed in air, stable voltage and current could be detected. While dipping the sandwiched device in bulk water, inversely, the current disappears and an obvious voltage can be detected. Without filter paper as the separator, the voltage disappears and certain current can be kept in air. Upon dipping in water, both signals of voltage and current disappear. By comparing device structures (1), (2), and (3), it is found that a voltage is generated when there is separator between Zn and carbon cloth under the existence of water, indicating that the voltage is from the potential of Zn sheet and carbon cloth. By comparing device structures (2), (3), and (4), it is found that the current only exists when exposed in air, which indicates that the current generation may be related to water evaporation and reaction with oxygen on the carbon cloth surface.^[^ ^4^, ^35^, ^36^ ^]^

To determine the relationship between current generation and water evaporation, the current on the carbon cloth surface under different environment is measured, as shown in Figure 2f (I–VI). In schematic I, there is no current can be detected without dropping water. In schematic II, 0.61 uA of current is generated after dropping water. The current increases to 1.22 uA under the assistance of light illumination from solar simulator in schematic III. However, no current is detected when dipping carbon cloth in water in schematic IV. Further, as shown in V and VI, there is no current on the surface of carbon cloth without water when heating it. Inversely, a current of 4.21 uA is observed with water under heating. Based on above results, it can be concluded that water evaporation is responsible for generating current, and the magnitude of current is related to the velocity of water evaporation. The faster evaporation induces bigger current, which excludes thermoelectric effect. The origination of output current should be the evaporation potential caused by water evaporation on the carbon cloth surface. The change of free energy generated by evaporation plays a major role in power generation.^[^ ^37^ ^]^

Figure 2g shows the variation of voltage and current with different loading resistances. A peak output density of 142.72 uW cm^−2^ could be achieved at 900 Ω as show in Figure 2h.^[^ ^38^ ^]^ Figures S5 and S6 (Supporting Information) show the curves of the voltage and current values as a function of time when changing the value of the load resistance. Replacing the filter paper separator of the device with a 2 mm cell diaphragm, the device performance is shown in Figure S7 (Supporting Information). It can be found that the output current density values of the device with the cell diaphragm are reduced. To investigate the reason for this, a carbonized melamine sponge (CMF) layer is used to replace the diaphragm, the CMF is a skeleton of network structure as shown in Figure S8 (Supporting Information). The performance of the 2 mm cell diaphragm is similar with that of the 2 mm CMF, and the 1 mm CMF performs the best, which indicates that the design of porous structure for water wetting is important for achieving satisfied performance.^[^ ^39^, ^40^ ^]^ Replacing Zn plate with Al plate as shown in Figure S9 a–c (Supporting Information), two configurations of devices are designed and tested. With the carbon cloth on the top, the voltage is kept at ≈0.52 V with a current density of 24.46 µA cm^−2^. With the Al plate on the top, the voltage is kept at 0.59 V with a current density of 8.17 µA cm^−2^. Figure S10 (Supporting Information) is the I–t curve of the device with intermittent exposing in N_2_ and air, which shows similar behavior with that of Zn plate based device.

**Figure 3:**
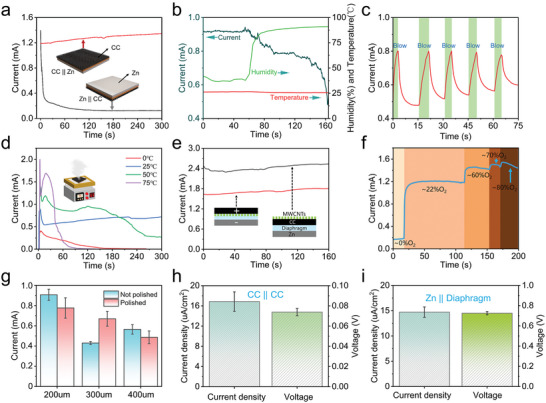
a) Output of current when the device is turned over; b) Current curve for a device with a change in humidity for normal operation; c) Current output for accelerated evaporation by gas flow from the device surface; d) Current curve after changing temperature for normal operation of the device; e) Current output of a device loaded with multi‐walled carbon nanotubes on one side of the carbon cloth with the distribution towards the filter paper and air; f) Current curve of Zn || CC device at different oxygen concentrations; g) Current output with and without Zn polishing; h) Current‐voltage output of the device with carbon electrodes; i) Zn self‐corrosion current voltage when Zn is in contact with the diaphragm.

To explore the potential mechanism of the device, further experiments were conducted. The device structure was flipped to measure the current, and the results showed that water evaporation had a significant impact on the device's performance as shown in **Figure** **3**a. In Figure 3b, the humidity of the device working environment was changed, and the device's current output gradually decreased with increasing humidity due to the hindered evaporation. Gas flow was used to accelerate evaporation, and the device's current output increased when there was a gas flow, while rapidly decreasing when the gas flow disappeared as shown in Figure 3c. The temperature of the environment also affected the device's current output, with higher temperatures resulting in faster evaporation and higher current outputs as shown in Figure 3d, when the temperature was increased from 25 °C to 50 °C, the output current of the device changed by ≈0.52 mA, which is labeled as ΔI _evaporative‐50°C_ = 0.52 mA, and when the temperature was increased from 25 to 75 °C, the output current of the device changed by ≈1.21 mA, which is labeled as ΔI _evaporative‐75°C_ = 1.21 mA. The device is immersed in a beaker filled with water to prevent evaporation. The temperature of water is controlled by a heater, and at the same time the output current of the device is monitored. Figure S11b (Supporting Information) shows a schematic of the device dipped in water and a heater at the bottom. The current output curve of the device in water with the increasing temperature is shown in Figure S11c (Supporting Information), when the temperature was increased from 25 to 50 °C, the output current of the device changed by only 0.05 mA, which is labeled as ΔI _Resistive‐50°C_ = 0.05 mA, and when the temperature was increased from 25 to 75 °C, the output current of the device changed by only 0.16 mA, which is labeled as ΔI _Resistive‐75°C_ = 0.16 mA. The results of ΔI _evaporative‐50°C_ > ΔI _Resistive‐50°C_ and ΔI _evaporative‐75°C_ > ΔI _Resistive‐75°C_ demonstrates that while the internal resistance of the device at elevated temperature could affect output, the reason of output increase with temperature is mainly due to the increase in evaporation rate. As shown in Figure 3e, multi‐walled carbon nanotubes were loaded on one side of the carbon cloth. When the carbon nanotubes faced the air, the current output was higher than when they faced the filter paper because the water molecules evaporated from the surface of the carbon nanotubes interacted more strongly with the carbon nanotubes than with the carbon cloth, demonstrating that the water evaporation on the surface of the carbon nanotubes affected the current output of the device. Furthermore, the presence of oxygen was found to have a direct effect on the device's output current in Figure 3f. Current testing on different Zn sheets, including both polished and unpolished ones in Figure 3g, showed that the unpolished Zn surface with an oxide film consisting of zinc oxide, zinc carbonate, and zinc hydroxide could prevent further oxidation of the internal zinc matrix and still produce the same performance. Additionally, replacing the Zn electrode with a carbon electrode also produced current output as shown in Figure 3h, but the OH^−^ generated by evaporation couldn't migrate efficiently due to the small potential difference between the two electrodes, leading to reduced current density output. Finally, submerging the device in water resulted in weak current output due to the self‐corrosion of the Zn as shown in Figure 3i, indicating that the device was severely affected by water evaporation and required deionized water with a pH = 7 to operate optimally. To further investigated the effect of the corrosion of the Zn electrode, a 10 nm Ag layer is deposited on Zn electrode. Figure S12a (Supporting Information) shows schematic diagram of a device with Ag deposited Zn electrode, where the Zn surface is coated with Ag to prevent direct contact between Zn and H_2_O. Figure S12b (Supporting Information) shows current and voltage curves of the device with Ag deposited Zn electrode, where the Ag film immobilizes the Zn atoms on the surface of the Zn sheet to prevent direct corrosion of Zn, and the active electrons on the Zn surface form a potential difference with CC through the Ag film. The evaporation device performance is still feasible with this structure of Zn/Ag ||CC. In this paper, the capacitance formed by the electrode potential between Zn and CC is used to store the OH^−^ generated by water evaporation; the Zn surface is coated with Ag to prevent direct Zn corrosion, retaining the characteristics of a large potential difference between Zn/Ag and CC, and the Zn in the device does not corrode but also generates a high current voltage, proving that the Zn corrosion current of the device is secondary. Figure S12(c) and (d) (Supporting Information) show the optical photographs of Pt electrode clamps with CC and Zn connections; Pt is an inert metal and does not undergo redox reactions with water at room temperature. The effect of diaphragm thickness on the output current was experimentally investigated, Figure S13a (Supporting Information) shows diagram of the different thicknesses of the diaphragm and Figure S13b (Supporting Information) shows current and voltage based on different diaphragm thicknesses, which shows that the 1 mm diaphragm has the highest current and the output current of the device gradually decreases as the thickness of the device diaphragm increases. This is because the increased thickness of the diaphragm increases the resistance and ion migration distance, thus reducing the current flow capability and resulting in a decrease in output current.

From the above experimental investigation, the following conclusions can be drawn: 1. the device voltage comes from the potential between the metal electrode and the carbon cloth; 2. the device has to be exposed to the air when outputting current; 3. the device current disappears and the voltage remains unchanged in nitrogen environment or immersed in water, which should be affected by oxygen molecules; 4. the faster the evaporation of water molecules on the carbon cloth surface, the greater the current is; 5 On the metal sheet, no substantial redox reactions were observed.

**Figure 4:**
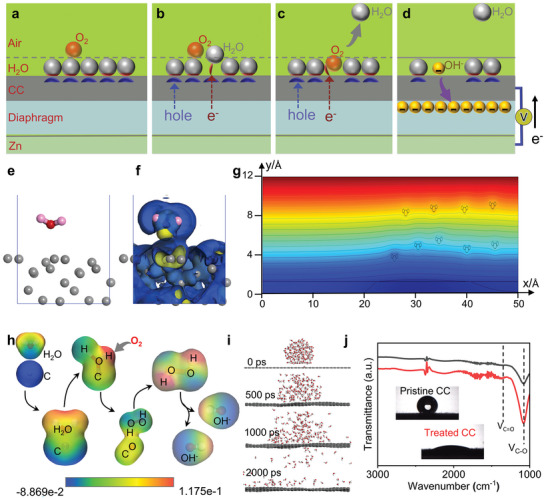
Schematic illustration of the electricity generation mechanism. a) Electron transfer from CC to H_2_O surface; b) Electrons on CC surface after H_2_O evaporation; c) Electrons with surrounding H_2_O and O_2_ make OH^−^; d) OH‐ migration generates current; e,f) Differential charge density diagram of water molecules adsorbed on carbon cloth surface; f,g) Electric field fluctuation on carbon cloth surface caused by water molecules; h) The process of OH^−^ formation by polarized adsorption of oxygen by water molecules; i) Simulation of evaporation process of 100 water molecular clusters; j) FTIR spectra of carbon cloth before and after hydrophilic treatment. The insets are the optical pictures of contact angle measurement.

To sum up, the working mechanism of this device may be that water molecules attract free electrons on the surface of carbon material, and loose electron clouds on the sp2 and sp3 hybrid orbits in the carbon material are gathered on the surface of carbon material, as shown in **Figure** **4**a a schematic diagram of the adsorption of H_2_O and O_2_ on the CC surface. The H_2_O molecules on the CC surface interact with the electron cloud on the CC surface, and part of the electrons on the CC surface are transferred to the H_2_O molecules.^[^ ^8^, ^41^, ^42^ ^]^ H_2_O molecules that have received extra electrons have higher energy than the surrounding H_2_O and evaporate more easily.^[^ ^43^, ^44^ ^]^ When the water molecules absorb heat and break the hydrogen bonds, they will evaporate into the air, and the extra electrons will be transferred to the surface of the CC instantaneously as the water evaporates, as shown in Figure 4b. The excited electrons caused by the evaporation of water molecules are instantly extracted by H_2_O and O_2_ on the CC surface to generate OH^−^, as shown in Figure 4c. The potential formed by Zn and CC causes OH^−^ to migrate under the electric field force to form a current, as shown in Figure 4d.

To understand the potential mechanism, we conducted first principle calculation of the interaction between carbon cloth and water molecules.^[^ ^45^, ^46^, ^47^, ^48^, ^49^, ^50^ ^]^ Figure 4e shows that the water molecules are adsorbed on the surface of the carbon cloth, and the carbon cloth adopts the disordered carbon atom model. Figure 4f shows that for water molecules absorbed on the surface of the carbon cloth, the surface charge of the oxygen atom shifts to the hydrogen atoms, and the water molecule is polarized. After the polarization, the charge on the surface of the hydrogen atom is coupled with the free electrons on the surface of the carbon layer, so that the free electrons on the carbon surface are transferred to a position farther from the surface of the carbon layer through the polarized water molecular orbital, which is conducive to interact with the oxygen molecules adsorbed on the water. Figure 4g shows electric field fluctuation on carbon cloth surface caused by water molecules. The blue represents high potential, and the X axis and Y axis are respectively the spatial position axis. X (0–20) is anhydrous surface, and X (20–50) is water included surface. From the potential contour, it can be seen that the electric field intensity on the side of water included surface is shifted upward, indicating that water molecules affect the electric field distribution on the surface of the carbon layer and have a coupling effect, which is consistent with the first principle calculation of the electron coupling interaction between the polarized water molecules and the carbon surface.^[^ ^31^, ^51^, ^52^ ^]^

Figure 4h explains the process of water molecules adsorbed on the surface of the carbon layer and polarized to form OH^−^ ions. The blue represents negative potential, and the red represents positive potential. When the water molecule closes to the C atom, the electron cloud overlap induces the polarization of water molecule, and the C atoms bond with the O atom. At the same time, the positive potential of H atom surface is enhanced, and the O_2_ molecule is adsorbed on the H site, causing the polarized water molecule to lose two H atoms and form H‐O‐H intermediate state, which is affected by the excited surface electrons of the carbon and the molecular thermal vibration, and finally decompose into OH^−^.^[^ ^53^, ^54^, ^55^ ^]^ Figure 4i is the simulation of evaporation process of 100 water molecular clusters at 40 °C. After 2000 ps, the water molecules have completely changed into the vapor. It can be seen that a layer of water molecules on the surface of the carbon layer has not evaporated away because the water molecules have enhanced their adsorption capacity with the carbon layer after polarization. The energy of the whole system increases with the increase of the heat absorption time, indicating the increased vibration of surface state electrons.^[^ ^56^, ^57^, ^58^, ^59^ ^]^ As shown in Figure S14 (Supporting Information), replacing the disordered carbon with graphene achieves the similar result with that of the disordered carbon. The closer the water molecule is to the graphene surface, the greater the influence on the electron distribution on the graphene surface. Figure 4j shows the FTIR spectra of the carbon cloth before and after hydrophilic treatment. After treatment, C═O and C─O stretching vibration are all enhanced, which is because the hydrophilic carbon cloth absorbs water molecules in the air and forms C═O and C─O bond after polarization.^[^ ^54^, ^60^, ^61^ ^]^

Figure S15 (Supporting Information) shows the process of generating OH^−^ ions and storing in electric double‐layer capacitor due to the interaction between oxygen and water molecules adsorbed on the surface of carbon materials from 3D and 2D perspectives.^[^ ^62^, ^63^ ^]^ The local aggregation of electrons caused by water evaporation provides support for the reaction of oxygen and water molecules, and the generated OH^−^ ions shuttle through the filter paper to supply power to the load devices. The charge‐discharge curve and Nyquist curve in Figure S16 (Supporting Information) also supports the conclusion. The constant current charge–discharge curve does not belong to the discharge platform of the zinc‐air battery and the resistance value of the Nyquist curves is much greater than the impedance value of the zinc‐air battery, which is consistent with the result of Figure 2c.

**Figure 5:**
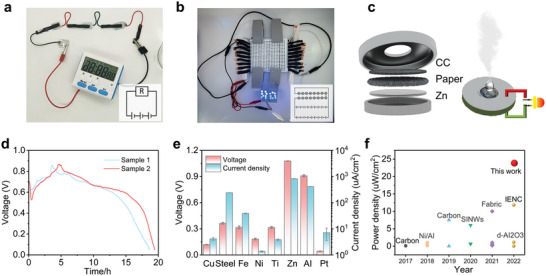
a) Three devices in series to drive a timer; b) Devices in series and parallel to drive 16 LEDs; c) Illustration of hydrovoltaic device structure; d) The V–t curve after dropping three droplets of water when discharging at 10 uA; e) The voltages and current densities achieved with different metal electrodes; f) Comparison of present work with previous reports.

Constant current discharge test is carried out on a single device as shown in Figure S17 (Supporting Information). After initial wetting stage, the voltage keeps stable at different discharging currents in the room temperature environment, indicating the stable output performance of the device. Dropping three droplets of water, the device could be powered continuously for 20 h as shown in Figure S18 (Supporting Information), and the decreases in voltage and current are due to the evaporation induced variation of water content. **Figure** **5**a shows three devices in series (the effective area of a single device is 0.6 cm^2^) to drive a timer. The experimental process is shown in Video S1 in (Supporting Information). The timer is steadily driven through a series connection of 8 devices, and the experimental process is shown in Video S2 in (Supporting Information). Figure 5b shows that the device can be integrated into a power supply to drive 16 LEDs, and the experimental process is shown in Video S3 in (Supporting Information). Individual device voltage and current measurement by multimeter, and the experimental process is shown in Video S4 in (Supporting Information). Figure 5c is the illustration of present device structure. A hole is opened on the top to facilitate the entry of water molecules and oxygen and the evaporation of water molecules. Figure 5d shows that after 3 droplets of water are added to the device, the device continues to discharge at a constant current of 10 µA for 18 h at room temperature.^[^ ^50^, ^64^ ^]^ The device finally stops working because of water evaporation loss. Eight devices are connected in series to drive two timers to work normally as shown in Figure S19 (Supporting Information). Figure 5e shows that various metal electrodes such as Cu, Steel, Fe, Ni, Ti, Zn, Al, Pt could also achieve current and voltage output, among which Zn and AI have the best performance.^[^ ^65^ ^]^ Because Zn and Al are active metals with high potential difference with carbon materials. The metal electrodes provide a source of stable voltage output for the device, but the current output depends on the water evaporation behavior. Zn and Al belong to active metals. During the research, it was found that there is a contribution of corrosion current in the output current of the device. Ti, Ni, Fe, Steel, and Cu belong to relatively inactive metals, and the potential difference between the device and CC is low, resulting in insufficient OH^−^ transmission power between the metal electrode and CC, and the output current of the device decreases, but there will also be weak corrosion. Pt is an inactive metal that can also generate weak currents and voltages with CC, demonstrating the universality of this device structure and the dependence on the metal electrode potential. In Figure 5f, we have shown that present device achieves the best record compared with previous reports.

## 3 Conclusions

In summary, we have demonstrated an asymmetric device composed of hydrophilic carbon cloth and Zn plate. It has been indicated that water and oxygen are continuously consumed to generate electrical energy, and the working behavior is originated from evaporating potential other than streaming potential. When the load resistance value of the device is 0.9 kΩ, the maximum power is 142.72 µW cm^−2^, which is the highest value among similar hydropower devices. It has been proven that other metals such as Cu, Al, and Fe et al. can also produce power output to be universal evaporating potential devices. The feasible structure and economic materials used in the device enable the possible commercial application of hydrovoltaic electrical energy generation devices.

## 4 Experimental Section

### Chemicals and Reagents

Carbon cloth (CC) were purchased from Ce Tech. Sulfuric acid (H_2_SO_4_), nitric acid (HNO_3_) was purchased from Sinopharm Chemical Regent. Zn, AI, Fe, Ni, Ti, and Cu were purchased from Wenghou Metal Material Firm, Hefei City. Pt sheet and Pt electrode clip were purchased from LEDONL AB.

### Acidification of Carbon Cloth

Typically, hydrophobic carbon cloth was dipped in 250 ml mixed solution of H_2_SO_4_ and HNO_3_ (H_2_SO_4_/HNO_3_ = 3/1, v/v) for 48 h. Then, it was washed by deionized water and dried.

### Fabrication of the Devices

Zn plates (2 cm*1 cm) were cleaned ultrasonically in alcohol and deionized water and dried at 70 °C. The acid carbon was sandwiched with Zn plate to form a device, which was separated by filter paper.

### Characterizations

Scanning electron microscopy (SEM) was used to investigate material structure (SEM, Zeiss MERLIN Compact). A Keithley 2400 was used to measure the current‐voltage curves. The CHI 660E electrochemical workstation was used to measure impedance, constant current charge/discharge, and cyclic voltammetry.

## Conflict of Interest

The author declare no conflict of interest.

## Supporting information

Supporting Information

Supplementary Video S1

Supplementary Video S2

Supplementary Video S3

Supplementary Video S4

## Data Availability

The data that support the findings of this study are available from the corresponding author upon reasonable request.
