# Supplementary material for: High Hydrovoltaic Power Density Achieved by Universal Evaporating Potential Devices
Source: Adv Sci (Weinh). 2023 Sep 15;10(30):2302941. doi: 10.1002/advs.202302941 (PMC10602524; doi:10.1002/advs.202302941)
Supplement: Supplementary file 1 — Supporting Information [file ADVS-10-2302941-s001.pdf]

ADVANCED SCIENCE
Open Access

Supporting Information

for *Adv. Sci.*, DOI 10.1002/advs.202302941

High Hydrovoltaic Power Density Achieved by Universal Evaporating Potential Devices

Fei Yu, Jialun Li, Yi Jiang, Liying Wang, Xijia Yang, Yue Yang, Xuesong Li, Ke Jiang, Wei Lü and Xiaojuan Sun**

Supplementary Information for

High hydrovoltaic power density achieved by universal evaporating potential devices

Fei Yu[1], *Jialun Li*[1], *Yi Jiang*[2], *Liying Wang*[1], *Xijia Yang*[1], *Yue Yang*[1], *Xuesong Li*[1], *Ke Jiang*[3], *Wei Lü*[1,3*], *Xiaojuan Sun*[3*]

[1]Key Laboratory of Advanced Structural Materials, Ministry of Education & Advanced Institute of Materials Science, Changchun University of Technology, Changchun 130012, People's Republic of China

[2]School of Science, Changchun Institute of Technology, Changchun, 130012, China

[3]State Key Laboratory of Luminescence and Applications, Changchun Institute of Optics, Fine Mechanics and Physics, Chinese Academy of Sciences, Changchun 130033, People's Republic of China

E-mail: lw771119@hotmail.com, sunxj@ciomp.ac.cn

Fax: +86-0431-85716426; Tel: +86-0431-85716421

* To whom all correspondence should be addressed.

Computational method. First-principles calculations were performed within the framework of DFT as encoded in Materials Studio Package code The Perdew−Burke−Ernzerhof (PBE) version of the generalized gradient approximation (GGA) was applied for the exchange and correlation. The van der Waals interactions were considered in the calculations, The kinetic energy cutoff of 300 eV was adopted for the wave function expansion. Moreover, Brillouin zone integration on the grid with a 2 × 2 × 1 k-grid mesh was performed for geometry optimization and calculation of density of states. The energy and force were converged to 2.0×10^{-5} eV atom^{-1} and 0.05 eV Å^{-1} to achieve high accuracy, respectively. A 14 Å vacuum layer thickness was applied to avoidvirtual interaction. The geometry was fully relaxed with the conjugate gradient method until the force on each atom was less than 0.05 eVÅ^{-1}.To elucidate the mechanism, Establish a disordered carbon model to adsorb water molecules, As shown in Figure 3d. And charge redistribution was defined as $\Delta\rho = \rho_{FG+w} - \rho_{FG} - \rho_{w}$, where ρ_{FG+w}, ρ_{FG} and ρ_{w} denote the charge distribution of the whole adsorption system, disordered carbon and water molecule.

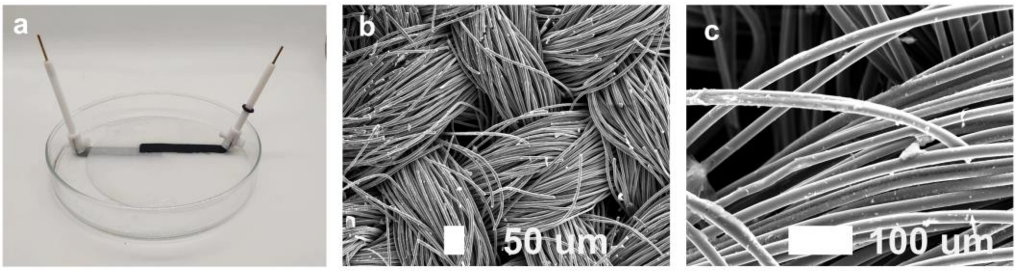

Supplementary Figures 1. (a) The photo of a unit device; (b) and (c) are SEM images of carbon cloth.

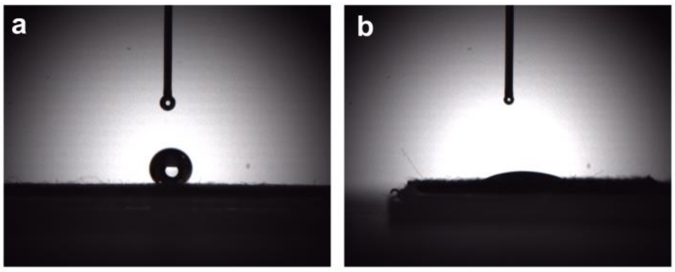

Supplementary Figures 2. (a) Optical photographs of the contact angle of hydrophobic carbon cloth; (b) Optical photographs of the contact angle of carbon cloth after acidfication.

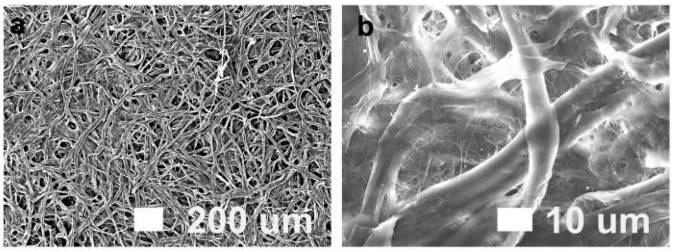

Supplementary Figures 3. (a) and (b) are SEM images of filter paper.

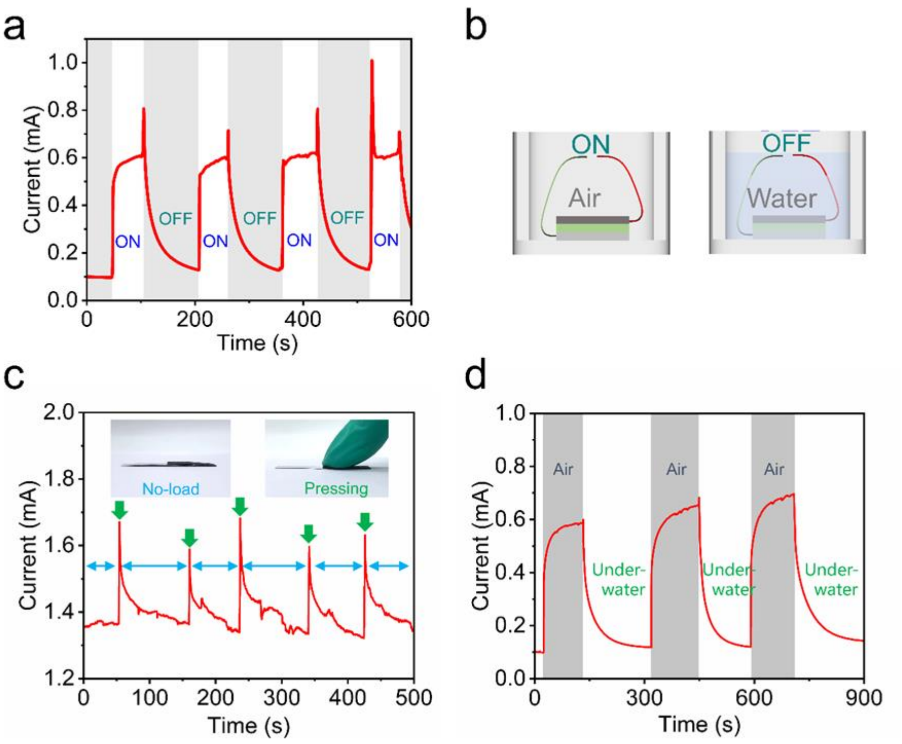

Supplementary Figures 4. (a) the current-time curve of the device submerged in water during injection. (b) the schematic diagram of the device submerged in water. (c) the current-time curve of the device when it is externally compressed. (d) the current variation of the slowly injected water-submerged device.

Figures 4a presents the current curve of the device when submerged in water and exposed to air, while Figures 4a is a schematic diagram of the device submerged in water during injection. When the device is submerged in water, the evaporation of

the water on the device surface stops, causing a decrease in the current, while placing the device in air allows for continuous evaporation, resulting in an increase in current. The appearance of a peak current in Figures 4a can be explained as follows: during the experiment, the device was placed in a culture dish and water was rapidly injected to immerse the device, achieving the goal of stopping evaporation. During the rapid injection of a large amount of water, the downward flowing water column had kinetic energy in the vertical direction and exerted an impact force on the device, similar to that of compression, causing an instantaneous deformation of the device and generating a current pulse peak. To prove this point, additional experiments were performed: (1) pressure-current tests were conducted on the device, and output current peaks were produced when the device was compressed, as shown in Figures 4c, demonstrating that the device's current output varies with pressure; (2) the device's water injection method was changed to a slow injection along the wall to reduce water wave impacts, and the data graph, as shown in Figures 4d, revealed the disappearance of the peak. In conclusion, the cause of the current peak formation is the influence of external forces on the device.

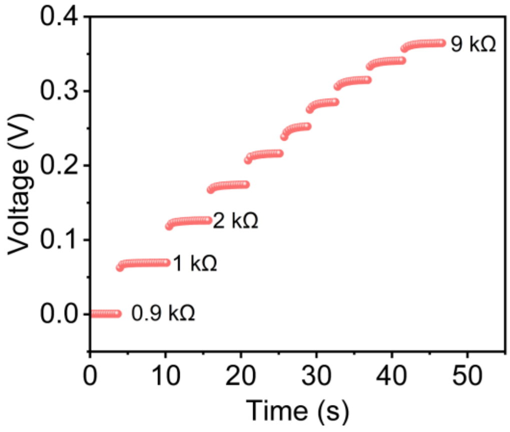

Supplementary Figures 5. The curves of the voltage as a function of time with different load resistances.

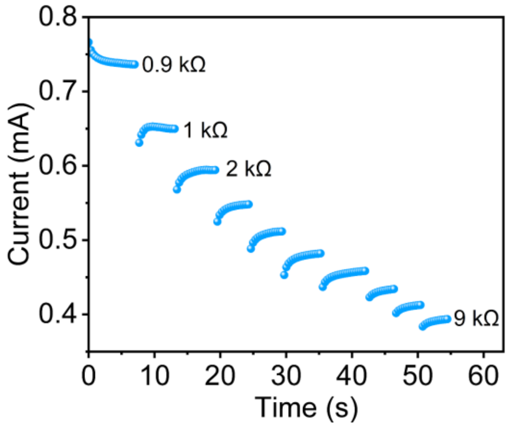

Supplementary Figures 6. The current values as a function of time with different load resistances.

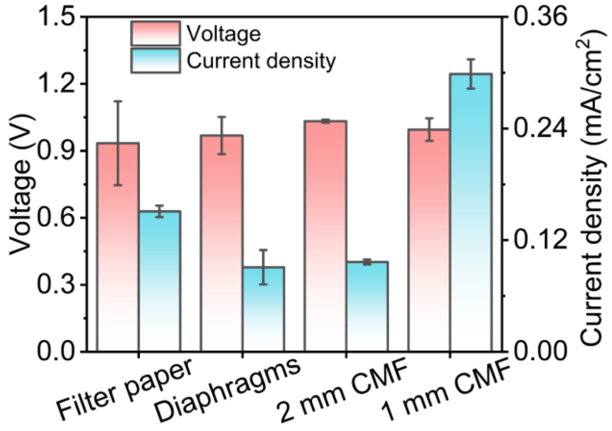

Supplementary Figures 7. The voltage and current density values of the device based

on different separators.

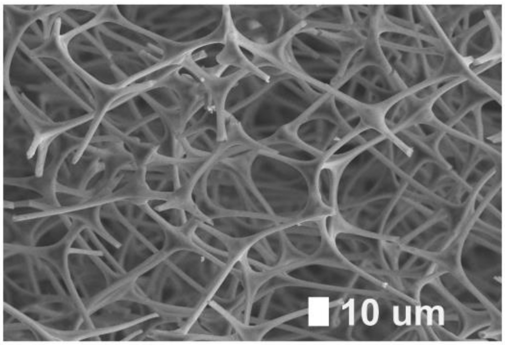

Supplementary Figures 8. SEM image of CMF.

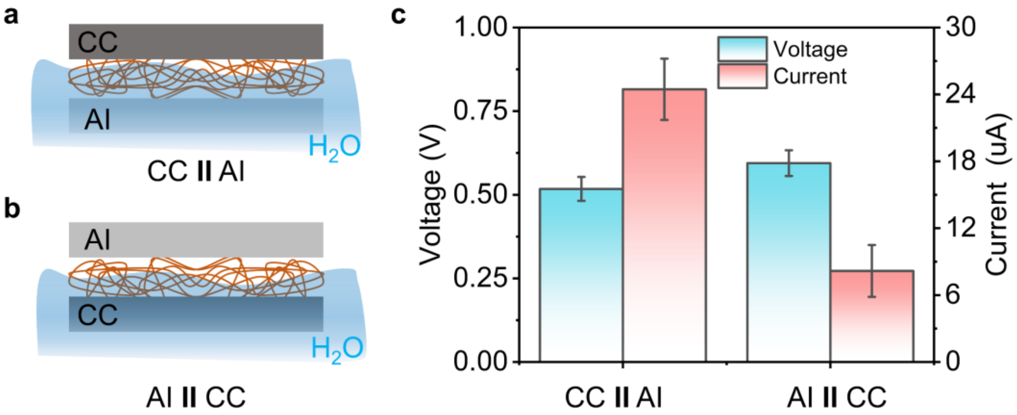

Supplementary Figures 9. Device structures by replacing Zn plate with Al plate. (a) The top is carbon cloth and the bottom is Al ;(b) The top is Al and the bottom is carbon cloth; (c) With structure in Figure a, the voltage is kept at 0.52 V with a current density of 24.46 μA/cm^2; With structure in Figure b, the voltage is kept at 0.59 V with a current density of 8.17 μA/cm^2.

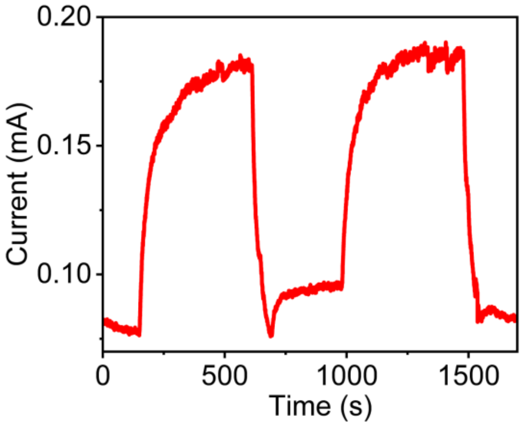

Supplementary Figures 10. The I-t curve of the device with intermittent exposing in N_2 and air, which shows similar behavior with that of Zn plate based device.

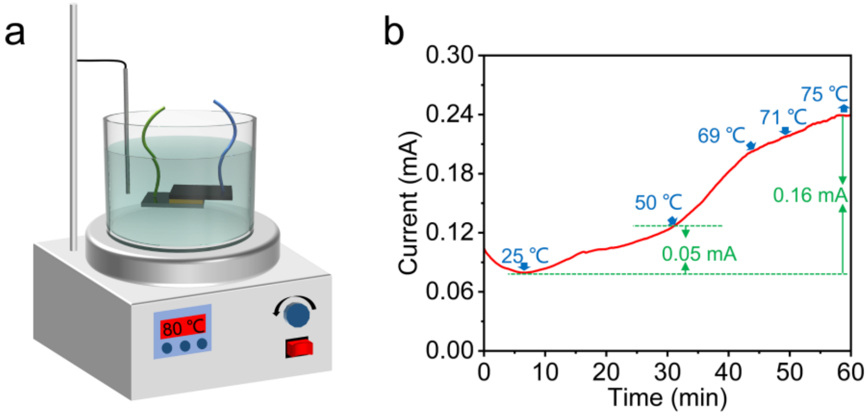

Supplementary Figures 11. (a) Schematic illustration of the device in hot water; (b) Current curve of the device after changing the water temperature.

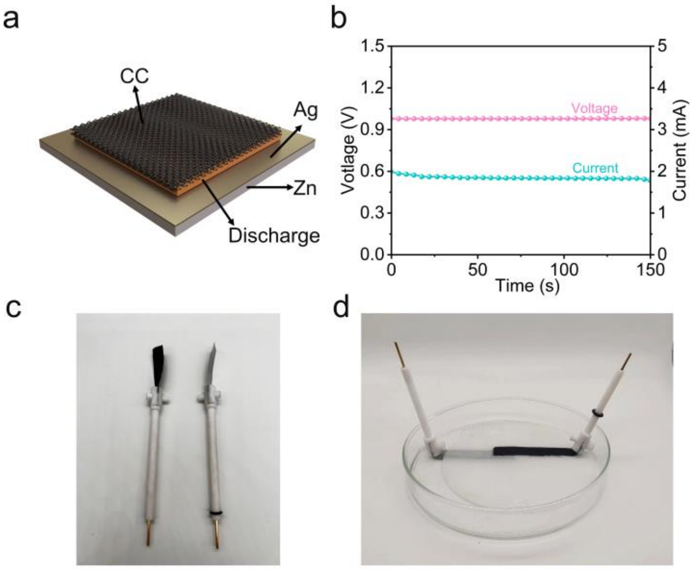

Supplementary Figures 12. (a) Schematic diagram of a device coated with Ag on a Zn electrode surface; (b) Current and voltage curves of device with Ag deposited Zn electrode; (c) and (d) are Optical photographs of testing the device with Pt electrode clamps.

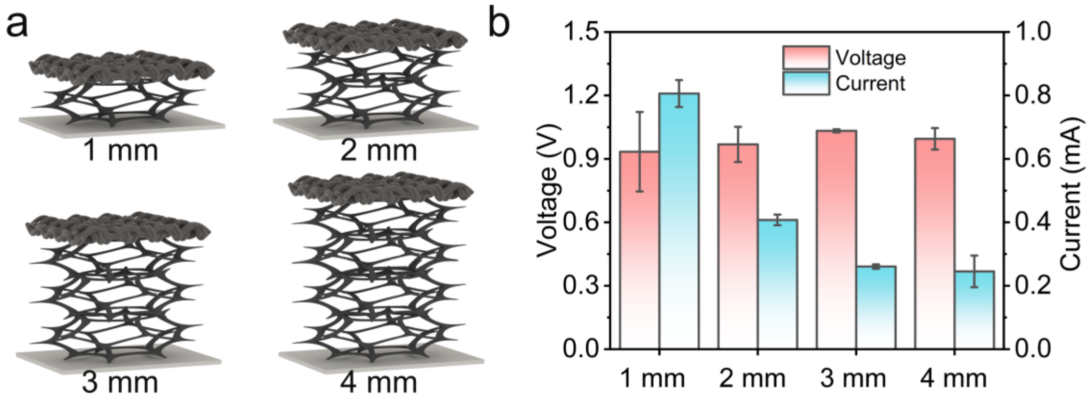

Supplementary Figures 13. (a) Diagram with different thicknesse; (b) Current and voltage tested based on different diaphragm thicknesses.

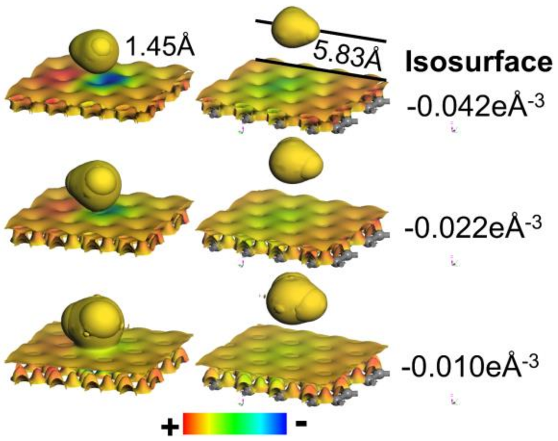

Supplementary Figures 14. Using graphene to replace disordered carbon, the deformation charge densities and equivalent surfaces are 0.042, 0.022, and 0.010, respectively. Simulation has shown that the small distances between the water molecules and the graphene surface has more strong effect on the charge distribution on the surface of the carbon material.

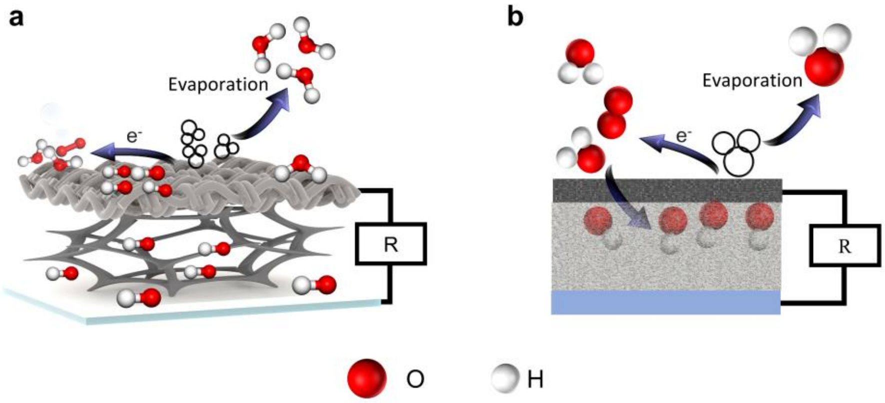

Supplementary Figures 15. The process of generating OH^- ions and storing in capacitor due to the interaction between oxygen and water molecules adsorbed on the surface of carbon materials from 3D and 2D perspectives.

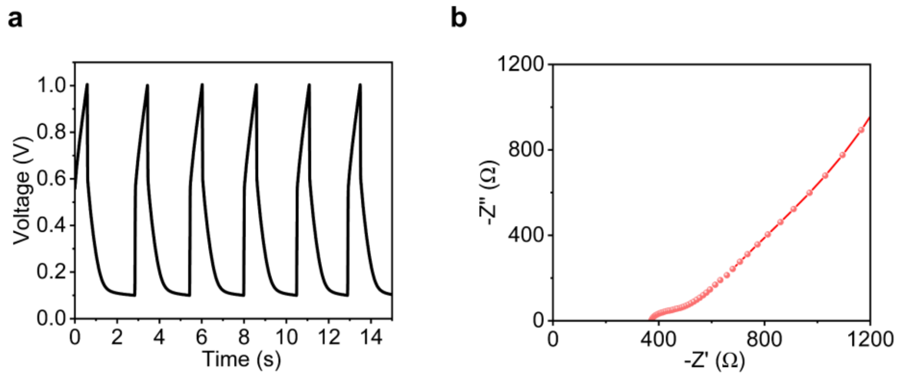

Supplementary Figures 16. (a) The charge-discharge curve. (b) The Nyquist curve.

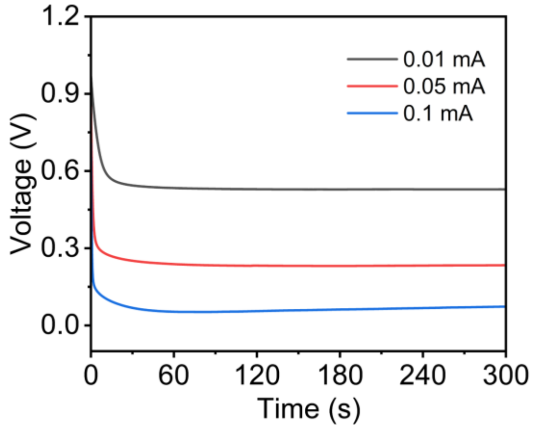

Supplementary Figures 17. Constant current discharge curves at different currents.

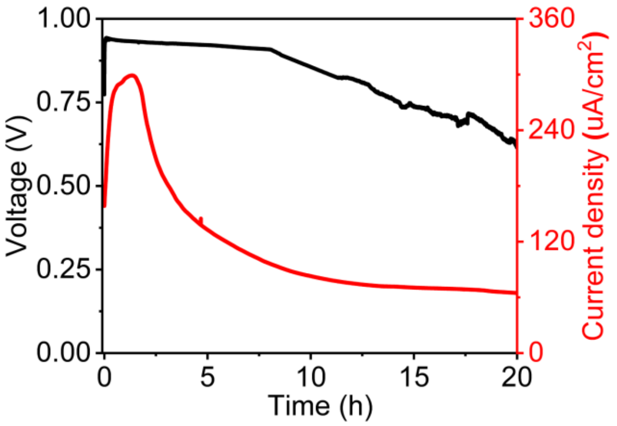

Supplementary Figures 18. The variation of voltage and current with time after dropping three droplets of water.

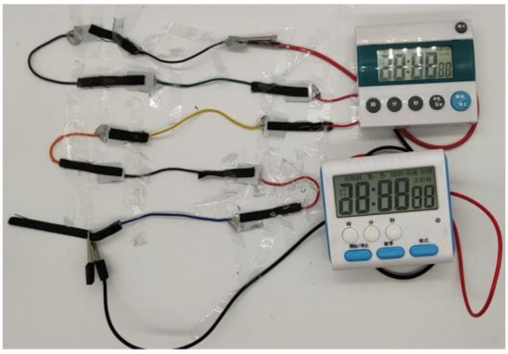

Supplementary Figures 19. Eight devices are connected in series to drive two timers.
